# Fears and anxiety disorders in young children with autistic disorders

**DOI:** 10.1192/j.eurpsy.2023.1533

**Published:** 2023-07-19

**Authors:** M. Kalinina, G. Kozlovskaya

**Affiliations:** 1 child pcychiatry; 2 child psychiatry, FSBSI MHRC, Мoscow, Russian Federation

## Abstract

**Introduction:**

Current level of mental health in children and adolescents is alarming, the precursors of it disorders can appear at an early age.

**Objectives:**

The phenomenological and prognostic features of children’s anxiety and phobic states were studied.

**Methods:**

The study conducted in 2022-2023 included 95 children of preschool age (among them 35 children under 3 years) with autistic disorders. For comparison, we used data from a follow-up study of 50 children lasting 15 years who had anxiety-phobic and autistic disorders at an early age. The control group included 30 children from the general population Assessment of the condition was carried out by clinical methods and original scales. PANSS scales were used in follow-up.

**Results:**

At an early age, these were fears of a protopathic nature, horror during sleep or during short-term sleepy states, the so-called “night terror”. Infants showed fears of touch, fear of manipulation: cutting hair, nails, pouring water while bathing in the shower, etc., due to a violation of sensory sensitivity. Also (among toddlers) frilly fears were found - water flowing into the bath, animalistic toys, along with autistic symptoms. In the catamnesis, there was an increase in symptoms. There were differentiated affective anxiety-phobic disorders, fragmentary phenomena and extended symptoms of a procedural nature (the total score on the PANSS scale exceeded 60 points, in 15 it reached 80). In the control group, normal development took place without psychopathology.

A direct relationship was found between the age of onset, the severity of early ontogenesis, and the phenomenological features of the disorders. In the premorbid of unfavorable forms of the course of schizophrenia, phobias were noted, comorbid outposts to symptoms of psychotic disorders resembling delusion, depersonalization symptoms.

**Conclusions:**

The findings suggest that phobic anxiety disorders in early childhood may be a precursor to mental illness later in life.

**Disclosure of Interest:**

None Declared

